# Myo-inositol improves the viability of boar sperm during liquid storage

**DOI:** 10.3389/fvets.2023.1150984

**Published:** 2023-07-26

**Authors:** Ali Jawad, Dongjin Oh, Hyerin Choi, Mirae Kim, Lian Cai, Joohyeong Lee, Sang-Hwan Hyun

**Affiliations:** ^1^Laboratory of Veterinary Embryology and Biotechnology (VETEMBIO), Veterinary Medical Center and College of Veterinary Medicine, Chungbuk National University, Cheongju, Republic of Korea; ^2^Institute of Stem Cell and Regenerative Medicine (ISCRM), Chungbuk National University, Cheongju, Republic of Korea; ^3^Graduate School of Veterinary Biosecurity and Protection, Chungbuk National University, Cheongju, Republic of Korea

**Keywords:** Myo-inositol, viability, boar, sperm, storage

## Abstract

**Introduction:**

Liquid preservation of boar semen is a highly preferred method for semen preservation in pig production. However, oxidative stress is the main challenge during the liquid preservation of boar semen in a time dependent manner. Therefore, supplementation of sperm with antioxidants during storage to protect them from oxidative stress has been the focus of recent research. Myo-inositol (Myo-Ins), the most active form of inositol, which belongs to the vitamin (Vit.) (B1 group has been shown to improve semen quality) (1). This study aimed to investigate whether Myo-Ins supplementation protects boar sperm in liquid preservation against oxidative stress and determine the appropriate concentration of Myo-Ins to be used in this regard.

**Methods:**

Boar sperm was diluted with a semen extender with different concentrations of Myo-Ins (2, 4, 6, and 8 mg/mL) depending on the previous studies (1, 24). Sperm motility and viability, plasma membrane and acrosome integrity, mitochondrial membrane potential (MMP), semen time survival, and gene expression were measured and analyzed on days 0, 1, 3, 5, and 7 for the different samples.

**Results:**

Different concentrations of Myo-Ins exerted different protective effects on the boar sperm quality. The addition of 2 mg/mL Myo-Ins resulted in higher sperm motility and viability, plasma membrane and acrosome integrity, MMP, and effective survival time. Investigation of mRNA expression patterns via qRT-PCR suggested that the 2 mg/mL Myo-Ins sample had increased expression of antioxidative genes.

**Conclusion:**

The addition of Myo-Ins to semen extender improved the boar semen quality by decreasing the effects of oxidative stress during liquid preservation at 17°C. Additionally, 2 mg/mL is the optimum inclusion concentration of Myo-Ins for semen preservation.

## 1. Introduction

Artificial insemination (AI) is the deliberate introduction of sperm into the cervix or uterine cavity of a female to achieve pregnancy (*in vivo* fertilization) through means other than sexual intercourse or *in vitro* fertilization (1). It is a fertility treatment for humans and a common practice in animal breeding, including pigs (1). Cryopreservation and liquid preservation are important methods for preserving the boar semen (1). Cryopreservation has been the main method for preserving semen for a long time. It is estimated that approximately 40% to 50% of boar spermatozoa do not survive after cryopreservation as they are highly sensitive to cold shock due to the plasma membrane composition having high unsaturated phospholipids levels and low cholesterol levels As a result of this, boar spermatozoa have a short lifespan when subjected to the freezing/thawing process because of their low survivability rates compared to other mammals (2). Therefore, an alternative method, liquid preservation of semen, is used to preserve boar semen for AI. Liquid preservation, is a simple and easy method to maintain high quality semen for a short duration, and is commonly used in pig production practices. However, the quality of boar semen declines rapidly; therefore, fresh boar semen cannot be stored for a long duration. The investigation of novel semen storage techniques may aid in extending the time and geographic boundaries of AI (2).

In the common boar sperm storage technique, sperm is stored at 17°C, which inhibits the movement of sperm and causes a reduction in the energy consumption so that the sperm can survive *in vitro* for longer (3). Despite the longer storage time, fertility and quality of boar sperm decline with time (4). Boar spermatozoa have high polyunsaturated fatty acid concentration in their plasma membranes and a low cholesterol/phospholipid ratio compared to the other animals semen making it more prone to oxidative stress (5). Therefore, boar spermatozoa are more prone to attack by reactive oxygen species (ROS), resulting in lipid peroxidation (6). *In vitro* storage of sperm leads to an increase in ROS production when certain conditions such as temperature are changed (7). Excessive ROS production reduces sperm motility, impairs cell integrity, and inhibits sperm-oocyte fusion (8). Previous studies have reported that adding antioxidants to semen extenders is an efficient way to protect boar sperm from oxidative stress *in vitro* (9).

Myo-inositol (Myo-Ins), the most active naturally existing form of inositol, and belongs to vitamin B1 complexes and is mainly produced in the human body by glucose-6-phosphate (1). In humans, supplementation of Myo-Ins *in vitro* protects sperm against oxidative stress to DNA and increases sperm motility and vitality in cryopreservation (10, 11). Myo-Ins play an essential role in various cellular activities such as cytogenesis, cell membrane formation, cell growth, morphogenesis, and lipid synthesis (12). Myo-Ins modulate intracellular calcium levels in the cellular signal transduction system by acting as a precursor for secondary messengers (13). Therefore, it plays a vital role in alterations in metabolism, sensitization of insulin, and especially in reproduction (14, 15). Myo-Ins levels in the seminiferous tubules were substantially higher than those in the serum (16). In human testicles, Sertoli cells are mainly involved in the production of Myo-Ins in response to follicle-stimulating hormone (FSH) (17). In addition, high concentrations of Myo-Ins monophosphatase-1 and Myo-Ins phosphate synthase in male reproductive organs promote the synthesis of Myo-Ins (18). Previous research was performed by Hinton and his collaborators in which he found out the concentration of Myo-Ins in luminal fluid of the testes and epididymis of different mammalian species in which boar sperm has a concentration of Myo-Ins 1.0–2.0 mM (19). Myo-Ins regulates many processes, including maturation, motility, capacitation, and the acrosomal reaction of spermatozoa (17), and is involved in the osmoregulation of seminal plasma (16). Furthermore, Myo-Ins is involved in improving the mitochondrial functions of sperm via Ca^+2^ ion influx that promotes the oxidation and generation of adenosine triphosphate (ATP), thereby condensing and preventing chromatin from apoptosis (18).

Myo-Ins was previously studied as an antioxidant agent *in vitro*/*in vivo* to treat infertility in men by improving spermatozoa quality and ultimately fertilization (20, 21). *In vitro* supplementation of Myo-Ins improved the sperm quality in oligoteratoasthenozoospermia (OAT) (18, 22) and the rate of fertilization with the embryo quality (21). Inositol plays a vital role in the protection of enzyme systems from lipid peroxidation and cryodamage and preserves the acrosomal integrity of ram post-thawed spermatozoa (23). *In vitro* supplementation of Myo-Ins in the freezing extender of frozen-thawed bull sperm improved sperm motility and other sperm parameters (24). Myo-Ins supplementation in the freezing extender protected cryopreserved dog sperm, attenuated sperm motility, kinematic parameters, membrane integrity, and curtailed chromatin damage and apoptosis (1). To date, there is a paucity of studies regarding the effects of Myo-Ins supplementation on boar semen during liquid preservation.

Therefore, this study aimed to investigate the effect of Myo-Ins supplementation on the motion characteristics (total motility, progressive motility and kinematic parameters), quality parameters [sperm viability, plasma membrane integrity, acrosome integrity, and mitochondrial membrane potential (MMP)], and gene expression (antioxidant genes) of boar semen during liquid preservation.

## 2. Materials and methods

### 2.1. Chemicals

Unless otherwise stated, all chemicals used in this study were purchased from Sigma Aldrich Co. (St. Louis, MO, United States). The LIVE/DEAD sperm viability kit and rhodamine 123 (Rd123) were purchased from Thermofischer Co. (Eugene, OR, United States). Semen extender (Duragen) was obtained from Darby Co. (Anseong, Gyeonggi-do, Republic of Korea).

### 2.2. Semen collection and transportation

The semen used in this experiment was commercial boar semen purchased from a semen sales company (Darby Co., Anseong, Gyeonggi-do, Republic of Korea) with no significant difference in vigor during all the seasons (25). Semen samples were packed in 90 mL commercial doses at a concentration of 3 × 10^9^ spermatozoa/dose and shipped to our laboratory within 1 h. After that high-quality sperm with >80% spermatozoa with normal morphology, approximately 80% motility, 60% progressive motility, 80% sperm viability, and 80% non-reacted acrosomes were kept at 17°C and used for further experiments. The fridge temperature was regularly monitored with a thermometer installed insides it. Thirty ejaculates were used in this study. The quality of spermatozoa was analyzed on day 0 (collection day) and on days 1, 3, 5, and 7 of preservation to evaluate total sperm motility, forward progressive motility, kinematic parameters, viability, membrane integrity, acrosome integrity, MMP, and gene expression.

### 2.3. Extender preparation

Duragen (Darby Co. Anseong, Gyeonggi-do, Republic of Korea), the long-term extender used in the present study, is composed of glucose 79.83 g, sodium citrate 7.07 g, diphasic potassium phosphate 3.54 g, tris 3.91 g, sodium bicarbonate 4.24 g, apramycin 0.59 g, and ampicillin 0.82 g/L in deionized water. In the treatment samples, we added Myo-Ins with the concentration of 2, 4, 6, and 8 mg/mL. Semen of the control group was stored in Duragen extender solution without adding Myo-Ins.

### 2.4. Semen processing

After collecting the fresh boar semen, the quality of the semen samples was evaluated, and all the semen samples were divided into five equal fractions. The control sample fraction was diluted with Duragen and the other fractions were diluted with 2, 4, 6, and 8 mg/mL of Myo-Ins. Lastly, all the semen samples were stored in a refrigerator at 17°C. All experiments were performed with at least four replicates for all the samples.

### 2.5. Sperm motility

**Figure 1:**
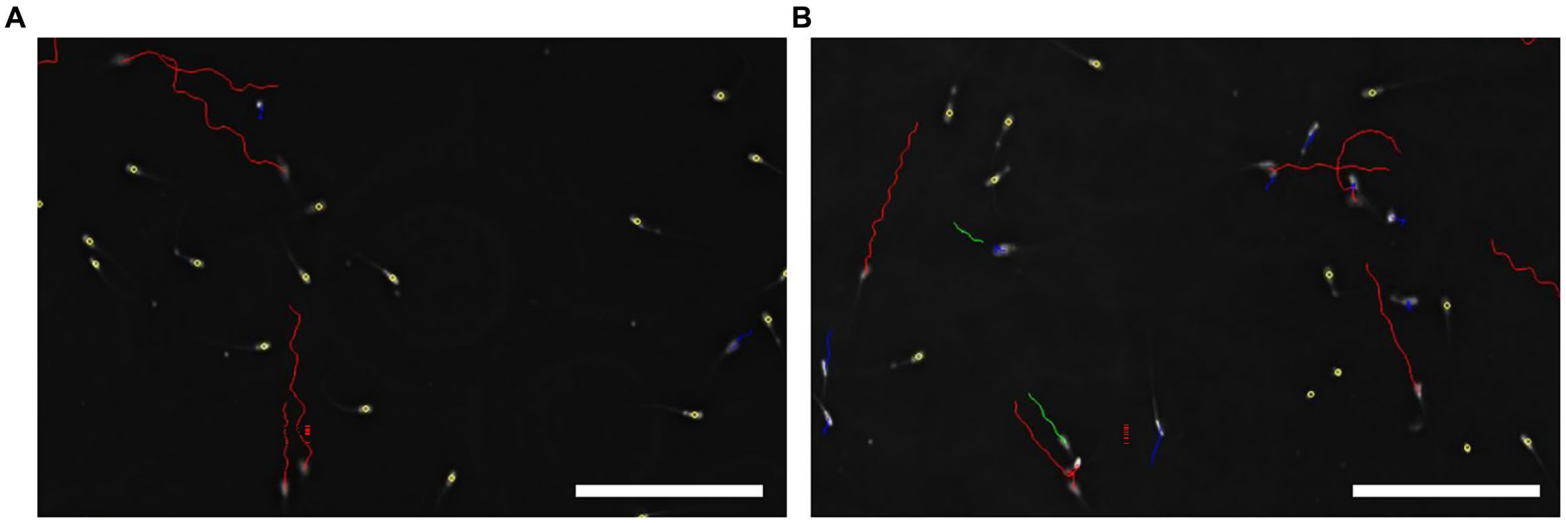
Representative image of sperm motility and trajectory paths captured with computer assisted semen analysis system (CASA). **(A)** Boar sperm motility in the control group. **(B)** Sperm motility in the treatment group (2 mg/mL) on day 7. Rapid, medium and slow movements are illustrated with red, green and blue colors, respectively. Immotile spermatozoa are indicated with yellow color. Scale bar = 100 μm.

Sperm motility was determined on days 0, 1, 3, 5, and 7 using a computer-assisted semen analysis system (CASA) (Sperm Class Analyzer, Microptic, Barcelona, Spain). In short, 2 μL of diluted semen was placed in a counting chamber (GoldCyto, Microptic, Spain) on a heated stage at 38°C. For each analysis, five fields were analyzed and at least 500 spermatozoa were counted. Motility patterns, including total sperm motility (TM), rapid progressive motility (RPM), medium progressive motility (MPM), immotility (IM), curvilinear velocity (VCL μm/s), straight line velocity (VSL μm/s), average path velocity (VAP μm/s), linearity [LIN = (VSL/VCL) × 100%], and straightness [STR = (VSL/VAP) × 100%], were measured as indicated in Figure 1.

### 2.6. Sperm viability

The viability of spermatozoa was assessed using the LIVE/DEAD sperm viability kit, as described by Yu and Leibo (26) with slight modifications. This was evaluated on days 0, 1, 3, 5, and 7. Basically, 5 μL of SYBR14 was added to (2.5 × 10^6^/mL)of spermatozoa, and 5 μL of propidium iodide (PI), and incubated in the dark for 5 min each at 37°C. Two smears were prepared from each sample, and smear-stained semen was used, air-dried, and observed under an epifluorescence microscope at 400× magnification. Approximately 300 spermatozoa were counted from each sample and classified as dead (red-stained) or live (green-stained) Spermatozoa with red and green fluorescence were recorded as percentages.

### 2.7. Plasma membrane integrity

The hypoosmotic swelling test (HOST) was performed to evaluate the functional membrane integrity of the sperm using the method described by Osinowo et al. (27). This was evaluated on the days 0, 1, 3, 5, and 7. In this assay, 30 μL of semen was mixed with a 300 μL hypo-osmotic solution (7.35 g of sodium citrate and 13.51 g of fructose in 1000 mL of distilled water with 150 mOsm/kg) (2). Sperm swelling was assessed after incubation at 37°C for 1 h, in which 10 μL of sperm was placed on a glass slide and observed at 400× magnification using a phase contrast microscope. A total of 300 spermatozoa were counted in at least five microscopic fields. Spermatozoa were classified as positive or negative based on the absence or presence of coiled tails spermatozoa with curled or swollen tails were recorded as percentages.

### 2.8. Acrosome integrity

The acrosome integrity of spermatozoa was assessed using a fluorescein isothiocyanate-conjugated peanut agglutinin (FITC-PNA) staining using a slightly modified procedure described by Yu and Leibo (26). This was evaluated on days 0, 1, 3, 5, and 7. Additionally, a glass slide smear using 10 μL of sperm was prepared and air dried for each sample. The smears were then fixed with absolute methanol at room temperature, stained with a FITC-PNA solution (100 μg/mL), and then spread with PBS. Subsequently, all stained smears were covered with parafilm for 20 min, dipped in distilled water for 15 min, and then air-dried. For each replicate, two slides from each sample were examined under an epifluorescence microscope. The percentage of fluorescent intact acrosomal spermatozoa with bright green fluorescence in the acrosomal region was counted with at least 300 spermatozoa per slide.

### 2.9. MMP assay

The mitochondrial activity of spermatozoa was assessed by Rd123 and PI staining using a previously described method (28). In this assay, 5 μL PI and 5 μL of Rd123 were added to the (1.25 × 10^7^/mL) spermatozoa and kept it in a dark dry heat block at 37°C for 15 min. At least 200 stained sperm cells were analyzed on each slide under a fluorescence microscope at 400× magnification. The sperm cells displaying red fluorescence in the head region were counted as dead, and the sperm cells showing a green mid-piece with no red heads were counted as viable sperm exhibiting functional mitochondria.

### 2.10. Quantitative reverse transcription-polymerase chain reaction

Expression of mRNA was analyzed using qRT-PCR for five specific genes with antioxidant functions, including nuclear erythroid factor 2 related factor 2 *(NRF2),* NAD(P)H dehydrogenase (quinone) 1 *(NQO1),* glutathione disulfide reductase *(GSR),* glutamate-cysteine ligase catalytic subunit *(GCLC),* and Kelch-like-ECH associated protein 1 *(KEAP1).* The primer sequences are listed in Table 1.

**Table 1:** Primers used for gene expression analysis.

mRNA	Primer sequences	Product size (bp)	Genbank accession number
*RN18S*	F: 5′-CGCGGTTCTATTTTGTTGGT-3′	219	NR_046261
	R: 5′-AGTCGGCATCGTTTATGGTC-3′		
*NRF2*	F: 5′-CCCATTCACAAAAGACAAACATTC-3′	75	XM_021075133
	R: 5′-GCTTTTGCCCTTAGCTCATCTC-3′		
*KEAP1*	F: 5′-AGCTGGGATGCCTCAGTGTT-3	100	NM_001114671
	R: 5′-AGGCAAGTTCTCCCAGACATTC-3′		
*GCLC*	F: 5′-GTTTTGTGAATCAGGACCCTA-3′	212	XM_003483635_4
	R: 5′-GCTTAGCTGAAGCTTTATTGC-3′		
*GSR*	F: 5′-TGGGCTCTAAGACGTCACTG-3′	106	XM_003483635
	R: 5′-TCTATGCCAGCATTCTCCAG-3′		
*NQO1*	F: 5′-ATGAACTTCAATCCCGTCAT-3′	191	NM_0011596
	R: 5′-CTCGGCAGGATACTGWGT-3′		

On day 7, control and Myo-Ins supplemented at 2 mg/mL sperm samples were stored at −80°C after washing with PBS. Trizol (TaKaRa Bio, Inc., Otsu, Shiga, Japan) was used for RNA extraction using the manufacturer’s instructions. SuperScript IV VILO Master Mix (Thermo Fisher Scientific, MA, United States) was used to convert the extracted RNA (1 μg of total RNA) into complementary DNA (cDNA). The synthesized cDNA was 2× SYBR Premix Ex Taq (TaKaRa Bio, Inc. Otsu, Shiga, Japan), and 5 pmol of specific primers (Macrogen, Inc., Seoul, Republic of Korea) were used for qRT-PCR. The mRNA expression was analyzed using the CFX96 Touch Real-Time PCR Detection System (Bio-Rad, Hercules, CA, United States). The cycling parameters were performed consecutively as follows: 95°C for 5 min, 40 cycles at 95°C for 15 s, 56°C for 15 s, and 72°C for 30 s. Relative quantification was performed by comparing the threshold cycle (Ct) at constant fluorescence intensity. Relative mRNA expression (R) was calculated using the equation *R* = 2^−[ΔCt sample – ΔCt control]^ (29). The R values were normalized to those of *RN18S* in the semen samples from each group.

### 2.11. Statistical analysis

All experiments in this study were conducted at least three times. Statistical analysis of data was done using GraphPad Prism (GraphPad Software, San Diego, CA, United States) and SPSS (version 12.0; SPSS, IBM, Armonk, NY, United States). Following the one-way analysis of variance test, Duncan’s multiple range test was used to examine percentage data (total motility, forward progressive motility, kinematic parameters, sperm viability, plasma membrane integrity, acrosome integrity, and MMP). An unpaired two-tailed student’s *t*-test was used to examine the average data (qPCR of day 7 sperm) from the two groups. Data are presented as mean ± standard error of the mean (SEM). Differences were considered statistically significant at *p* < 0.05.

## 3. Results

### 3.1. Effect of Myo-Ins on the total motility and forward progressive motility

The effects of different concentrations of Myo-Ins on boar sperm total motility (TM) and forward progressive motility (FPM) are shown in Table 2. Total motility of sperm was decreased from day 0 (87.4 ± 0.5%) with an increase in the preservation time day 7 (38.4 ± 1.5%) *in vitro*. Meanwhile, total motility of the sperm treated with 2 mg/mL Myo-Ins was higher than the other samples, and significantly (*p* < 0.05) improved on day 5 (58.6 ± 1.2%) and day 7 (48.9 ± 2.5%) as compared to the control sample. These results indicated that supplementation with 2 mg/mL Myo-Ins in the semen extender improved total sperm motility (Figure 1). Forward progressive motility of sperm gradually decreased from day 0 (72.9 ± 1.0%) to day 7 (27.4 ± 1.9%) in the control sample compared to the treatment sample. On day 3, in the 2 mg/mL treated sample, forward progressive motility was (55.7 ± 3.6%) compared with the control group which was (48.5 ± 1.8%) increased significantly (*p* < 0.05). However, on days 5 and 7, the FPM in the control group was (37.3 ± 2.6%) and (27.4 ± 1.9%) compared with the 2 mg/mL treated sample in which the FPM was (44.5 ± 1.8%) and (33.4 ± 2.4%). FPM on days 5 and 7 improved in the 2 mg/mL treated sample but not significantly compared with the control sample.

**Table 2:** Effect of Myo-Ins on boar sperm total motility and progressive motility.

Parameter	Treatment (mg/mL)	Time of storage (days)				
		0	1	3	5	7
Total motility (%)	0	87.4 ± 0.5	82.8 ± 1.8	62.8 ± 3.1^ab^	50.2 ± 2.3^b^	38.4 ± 1.5^b^
	2	87.4 ± 0.5	84.1 ± 1.0	70.5 ± 4.3^a^	58.6 ± 1.2^a^	48.9 ± 2.5^a^
	4	87.4 ± 0.5	83.2 ± 0.6	66.0 ± 2.4^ab^	55.9 ± 2.3^ab^	45.5 ± 3.7^ab^
	6	87.4 ± 0.5	84.5 ± 1.4	64.5 ± 1.5^ab^	52.5 ± 3.3^ab^	44.7 ± 4.2^ab^
	8	87.4 ± 0.5	83.8 ± 1.9	60.1 ± 1.6^b^	50.9 ± 2.7^ab^	42.9 ± 1.8^ab^
Forward progressive motility (%)	0	72.9 ± 1.0	70.0 ± 2.8	48.5 ± 1.8^b^	37.3 ± 2.6	27.4 ± 1.9
	2	72.9 ± 1.0	68.3 ± 2.3	55.7 ± 3.6^a^	44.5 ± 1.8	33.4 ± 2.4
	4	72.9 ± 1.0	68.9 ± 1.7	48.3 ± 1.1^b^	41.8 ± 3.6	30.8 ± 3.9
	6	72.9 ± 1.0	64.7 ± 2.8	45.3 ± 2.5^bc^	39.9 ± 4.1	27.9 ± 3.2
	8	72.9 ± 1.0	63.3 ± 2.2	40.5 ± 1.6^c^	37.9 ± 2.9	30.9 ± 1.9

^a–c^Values in the column with different superscript letters differed significantly (*p* < 0.05).

### 3.2. Effect of Myo-Ins on kinematic parameters

Evaluation of spermatozoa kinematic parameters showed that Myo-Ins supplementation significantly improved VSL (μm/s) and STR (%) parameters (Table 3). On days 1 and 7, VSL kinematic parameter significantly improved in the 8 mg/mL treated sample (79.0 ± 5.4) (42.7 ± 2.8) compared with the control group (57.2 ± 2.4) (28.8 ± 3.7). VSL significantly increased in 6 mg/mL treated sample (45.2 ± 4.9) compared with the control sample (35.6 ± 1.8) on day 5. Additionally, Myo-Ins supplementation in the 8 mg/mL sample on days 1, 3, 7 significantly improved the STR (61.8 ± 2.3), (61.9 ± 3.2), (75.7 ± 1.4) compared with the control sample (51.1 ± 3.5), (53.2 ± 1.8) and (63.5 ± 3.8). Moreover, STR also increased significantly in the 6 mg/mL on days 3 (63.0 ± 0.9) and 7 (76.1 ± 4.9) compared with the control sample (53.2 ± 1.8) (63.5 ± 3.8). On day 1, VCL (μm/s) parameter was higher at the 4 mg/mL sample (163.8 ± 3.7) compared to the control sample (142.4 ± 9.8). On day 5, 6 mg/mL sample (73.9 ± 8.6) showed the highest VCL compared to control sample (64.7 ± 5.7); however, on day 7, 8 mg/mL sample (64.7 ± 4.0) had the highest VCL kinematic parameter compared to control group (53.4 ± 2.1). Myo-Ins supplementation improved the other kinematic parameters assessed by CASA; however, there were no significant differences between the treated and control samples (Table 3).

**Table 3:** Effect of Myo-Ins on boar sperm kinetic parameters.

Parameter	Treatment (mg/mL)	Time of storage (days)				
		0	1	3	5	7
VAP (μm/s)	0	122.1 ± 3.2	113.9 ± 7.9	87.1 ± 17.1	53.7 ± 4.4	44.1 ± 3.7
	2	122.1 ± 3.2	115.9 ± 8.8	91.6 ± 18.2	58.3 ± 3.7	48.4 ± 3.7
	4	122.1 ± 3.2	135.2 ± 3.2	86.7 ± 16.9	59.6 ± 5.7	47.6 ± 3.4
	6	122.1 ± 3.2	119.2 ± 7.4	76.3 ± 9.5	59.9 ± 6.4	42.4 ± 4.2
	8	122.1 ± 3.2	119.7 ± 5.7	75.3 ± 11.8	54.5 ± 3.7	51.9 ± 3.4
VCL (μm/s)	0	149.3 ± 4.0	142.4 ± 9.8	109.8 ± 19.9	64.7 ± 5.7	53.4 ± 2.1^ab^
	2	149.3 ± 4.0	145.3 ± 11.1	108.2 ± 18.7	71.0 ± 5.0	61.3 ± 3.9^ab^
	4	149.3 ± 4.0	163.8 ± 3.7	104.0 ± 19.1	71.4 ± 7.2	60.4 ± 3.6^ab^
	6	149.3 ± 4.0	147.2 ± 7.9	93.5 ± 9.6	73.9 ± 8.6	52.1 ± 4.0^b^
	8	149.3 ± 4.0	139.8 ± 3.5	89.8 ± 13.5	64.5 ± 3.7	64.7 ± 4.0^a^
VSL (μm/s)	0	60.0 ± 1.7	57.2 ± 2.4^c^	47.2 ± 10.2	35.6 ± 1.8^b^	28.8 ± 3.7^b^
	2	60.0 ± 1.7	58.5 ± 3.6^c^	50.5 ± 13.2	36.8 ± 1.7^ab^	33.2 ± 1.5^ab^
	4	60.0 ± 1.7	73.1 ± 1.0^ab^	54.0 ± 12.9	42.9 ± 1.3^ab^	36.7 ± 4.7^ab^
	6	60.0 ± 1.7	67.0 ± 4.1^bc^	53.6 ± 7.9	45.2 ± 4.9^a^	34.9 ± 5.0^ab^
	8	60.0 ± 1.7	79.0 ± 5.4^a^	50.2 ± 7.3	43.2 ± 2.8^ab^	42.7 ± 2.8^a^
STR (%)	0	50.2 ± 1.1	51.1 ± 3.5^b^	53.2 ± 1.8^b^	65.7 ± 3.8	63.5 ± 3.8^b^
	2	50.2 ± 1.1	51.9 ± 3.5^b^	51.9 ± 2.4^b^	61.7 ± 2.3	66.9 ± 1.8^ab^
	4	50.2 ± 1.1	51.4 ± 0.6^b^	56.6 ± 1.4^ab^	70.1 ± 5.2	69.2 ± 4.3^ab^
	6	50.2 ± 1.1	57.3 ± 2.2^ab^	63.0 ± 0.9^a^	70.3 ± 3.9	76.1 ± 4.9^a^
	8	50.2 ± 1.1	61.8 ± 2.3^a^	61.9 ± 3.2^a^	73.3 ± 2.0	75.7 ± 1.4^a^
LIN (%)	0	41.8 ± 1.1	41.5 ± 3.5	45.5 ± 2.1	57.4 ± 4.1	54.3 ± 4.4
	2	41.8 ± 1.1	41.5 ± 3.1	45.4 ± 2.9	52.2 ± 2.5	55.8 ± 1.9
	4	41.8 ± 1.1	42.2 ± 0.8	48.3 ± 1.4	59.1 ± 5.0	55.9 ± 4.5
	6	41.8 ± 1.1	46.5 ± 2.1	53.9 ± 3.9	57.9 ± 4.6	63.8 ± 5.4
	8	41.8 ± 1.1	50.9 ± 3.7	52.5 ± 5.4	62.2 ± 2.2	61.9 ± 1.6

^a–c^Values in the column with different superscript letters differed significantly (*p* < 0.05). All the experiments are conducted with at least four replicates. VAP, average path velocity (μm/s); VSL, straight linear velocity (μm/s); VCL, curvilinear velocity (μm/s); STR, straightness [(VSL/VAP) × 100]; LIN, linearity [(VSL/VCL) × 100].

### 3.3. Effect of Myo-Ins on the sperm viability

**Figure 2:**
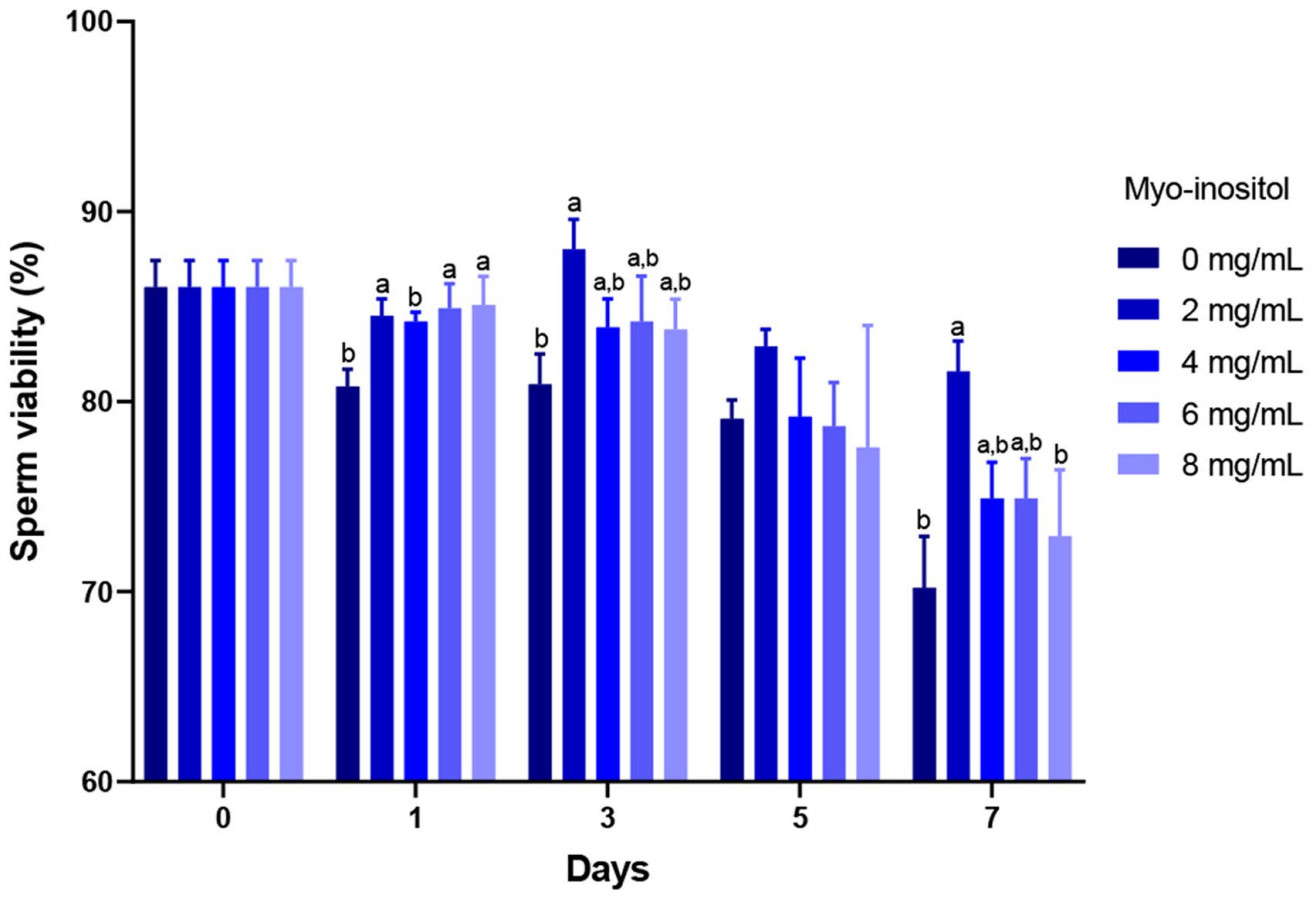
Effects of different concentrations of Myo-Ins on sperm viability sperm viability of boar semen during the liquid preservation. Within each endpoint, bars with different letters (a,b) indicate significant differences (*p* < 0.05) at various Myo-Ins concentrations For graph, all the values represent mean ± SEM. The experiments are replicated six times.

The effects of different concentrations of Myo-Ins on boar sperm viability during liquid preservation are presented in Figure 2. On day 1, sperm viability of the samples treated with 2 (84.5 ± 0.9%), 6 (84.9 ± 1.3%), and 8 mg/mL (85.1 ± 1.5%) Myo-Ins treated samples increased significantly compared to the control sample (80.8 ± 0.9%). On day 3 (88.0 ± 1.6%) and 7 (81.6 ± 1.6%), 2 mg/mL treated sample showed significantly higher sperm viability compared to the control sample (80.9 ± 1.6%) and (70.2 ± 2.7%). However, no significant differences were observed at 4, 6, and 8 mg/mL on days 3, 5, and 7 as shown in Figure 2.

### 3.4. Effect of Myo-Ins on the sperm plasma membrane integrity

**Figure 3:**
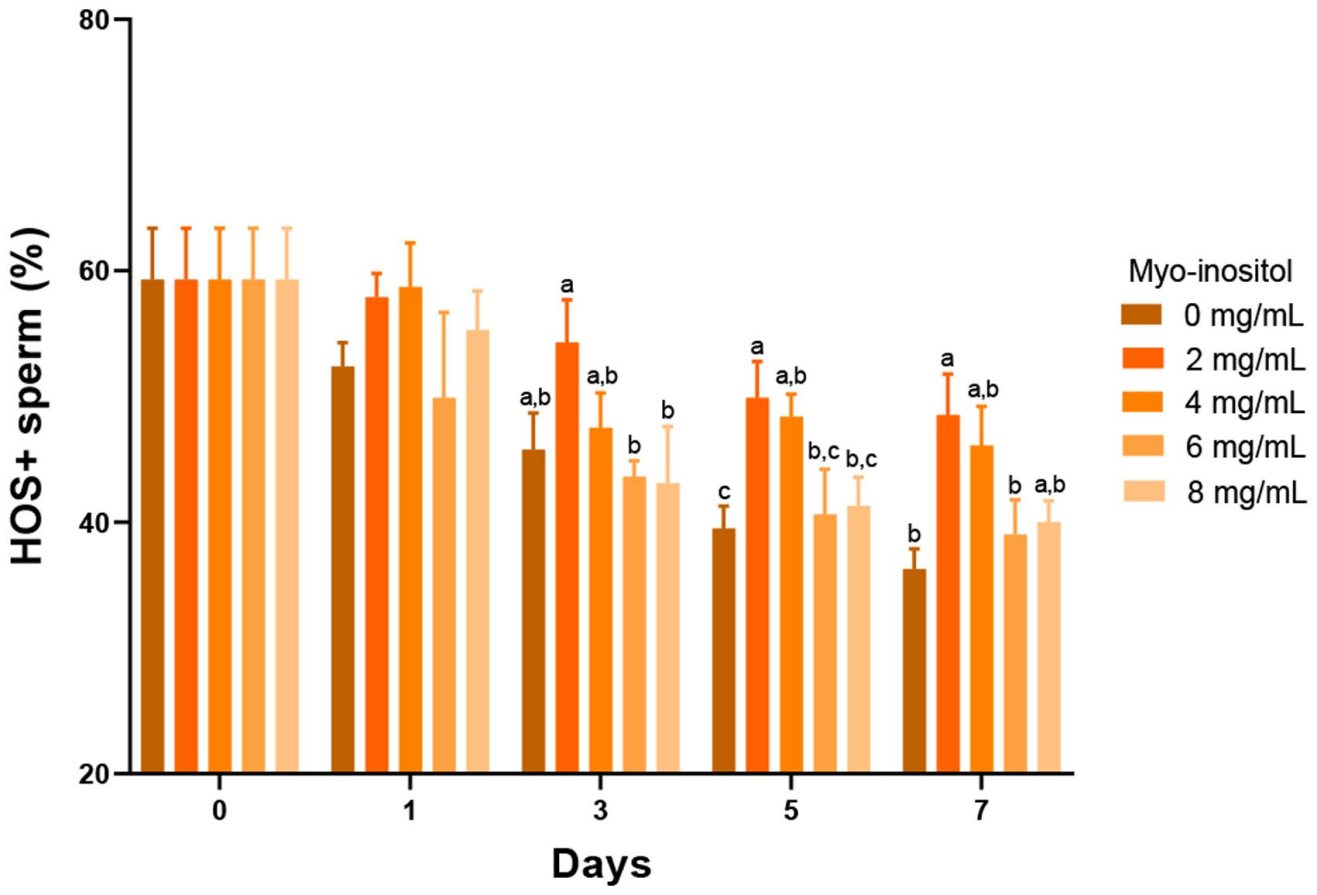
Effects of different concentrations of Myo-Ins on plasma membrane integrity Plasma membrane integrity of boar semen during liquid preservation. Within each endpoint, bars with different letters (a,b,c) indicate significant differences (*p* < 0.05) at various Myo-Ins concentrations For graph, all the values represent mean ± SEM. The experiments are replicated six times.

The effects of different Myo-Ins concentrations on boar sperm plasma membrane integrity during liquid preservation are shown in Figure 3. All treatment samples (2, 4, and 8 mg/mL) showed higher plasma membrane integrity than the control sample. After 3 days of preservation, significant results were observed on day 5 (49.9 ± 2.9%) (48.4 ± 1.8%) in the samples treated with 2 and 4 mg/mL compared to the control sample (39.5 ± 1.8%). In addition, on day 7 in the sample treated with 2 mg/mL plasma membrane integrity was significantly improved (49.9 ± 4.3%) as compared to the control sample (38.6 ± 0.8%). However, plasma membrane integrity did not improve significantly in the other treatment samples as illustrated in the Figure 3.

### 3.5. Effect of Myo-Ins on the sperm acrosome integrity

**Figure 4:**
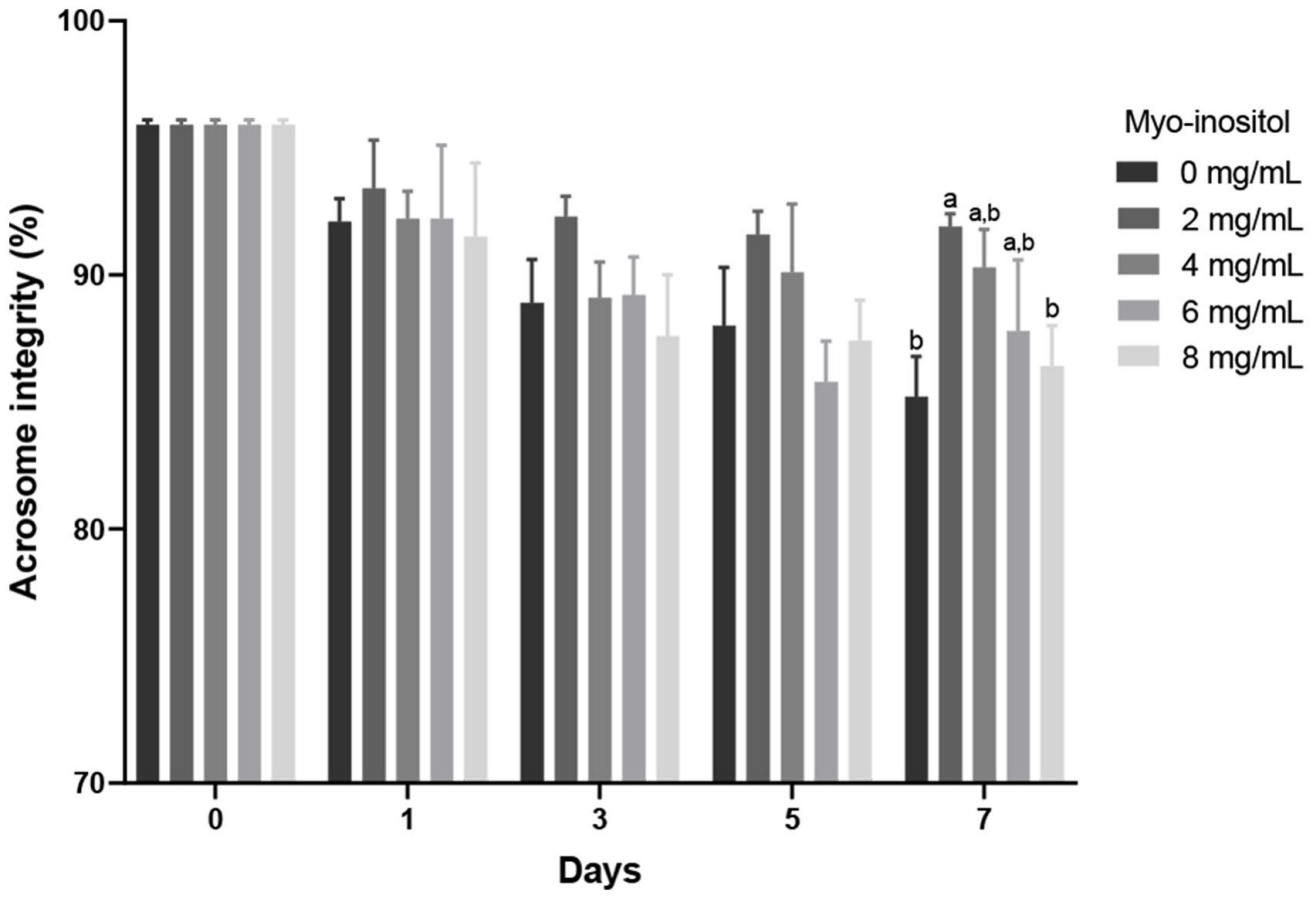
Effects of different concentrations of Myo-Ins on acrosome integrity Acrosome integrity of boar semen during liquid preservation. Within each endpoint, bars with different letters (a,b) indicate significant differences (*p* < 0.05) at various Myo-Ins concentrations For graph, all the values represent mean ± SEM. The experiments are replicated six times.

The effects of different Myo-Ins concentrations on boar sperm acrosome integrity during liquid preservation are shown in Figure 4. Acrosome integrity gradually decreased in all samples from day 0 to day 7; however, no significant difference was observed in the treated samples compared to the control sample. After 7 days of preservation, 2 mg/mL treated sample (91.9 ± 0.5%) showed significantly (*p* < 0.05) higher acrosome integrity compared to the control sample (85.2 ± 1.6%) as indicated in Figure 4.

### 3.6. Effect of Myo-Ins on the sperm mitochondrial membrane potential

**Figure 5:**
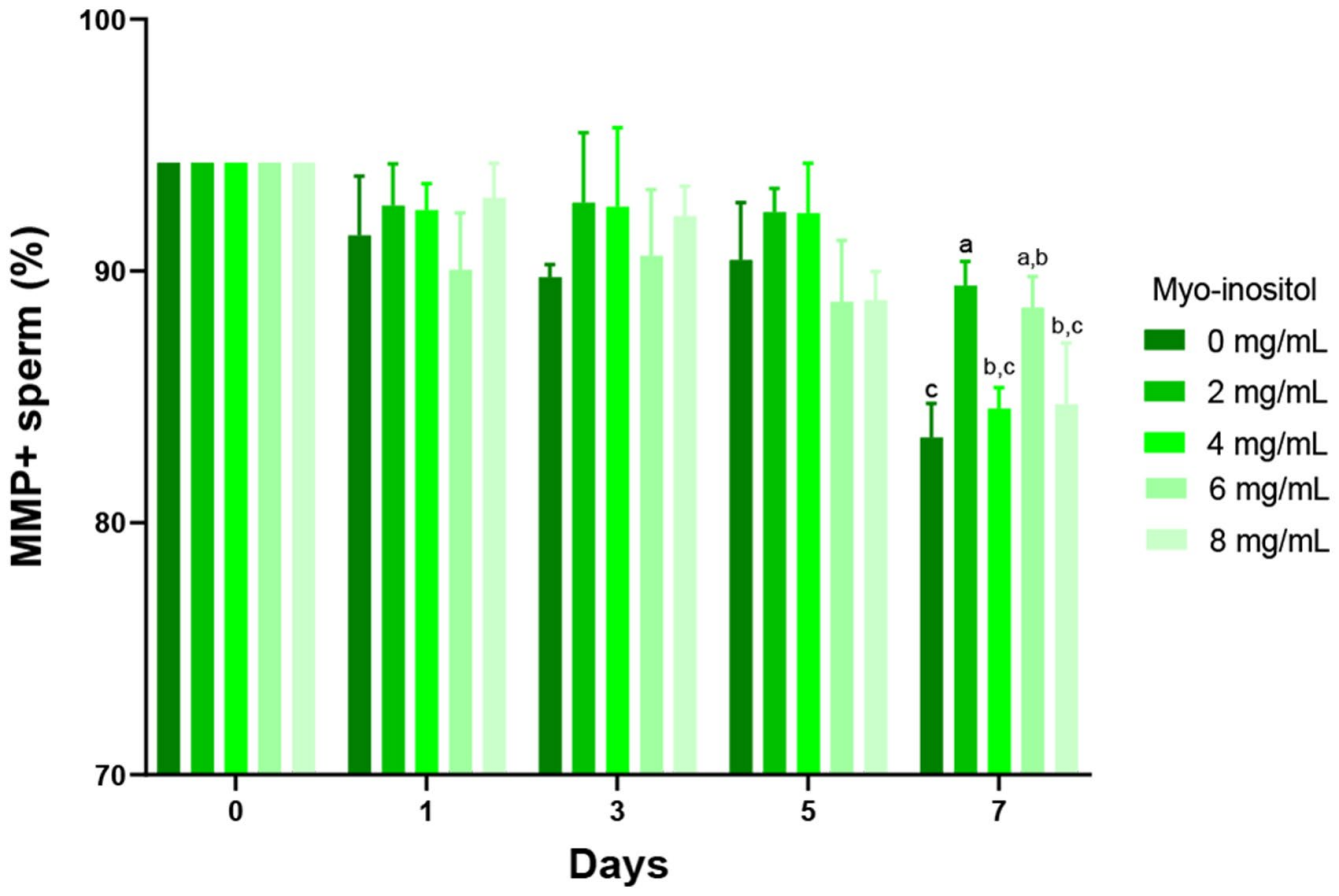
Effects of different concentrations of Myo-Ins on mitochondrial membrane potential (MMP) MMP of boar semen during liquid preservation. Within each endpoint, bars with different letters (a,b,c) indicate significant differences (*p* < 0.05) at various Myo-Ins concentrations. For graph, all the values represent mean ± SEM. The experiments are replicated four times.

The effects of different concentrations of Myo-Ins on the mitochondrial activity of boar sperm during liquid preservation are shown in Figure 5. Rd123 was used to assess the mitochondrial activity of sperm cells and PI was used to stain dead spermatozoa. The Myo-Ins-treated samples showed higher MMP compared to the control sample in a time-dependent manner. In 2 (89.5 ± 1.4%) and 6 mg/mL (88.6 ± 1.2%) samples, the MMP was significantly higher (*p* < 0.05) on the day 7 compared to the control sample (83.4 ± 1.4%). In the 4 and 8 mg/mL samples, MMP tended to increase; however, it did not significantly increase with storage time (Figure 5).

### 3.7. Effect of Myo-Ins on the gene expression

**Figure 6:**
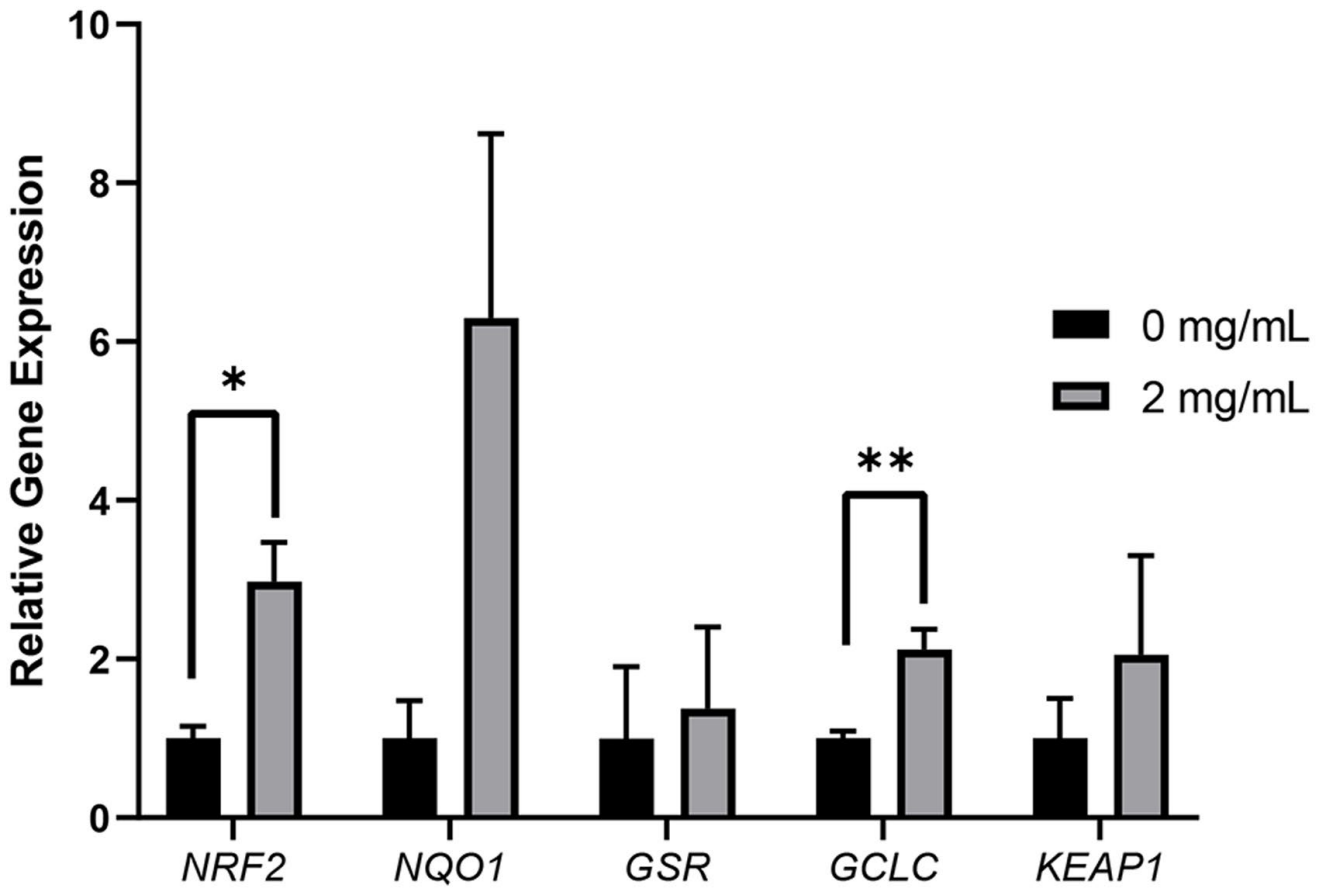
Relative mRNA expression levels of genes associated with oxidative stress. The mRNA expression levels of oxidative stress-related genes (*NRF2, NQO1, GSR*, *GCLC* and *KEAP1*) in sperm from the control and treatment (2 mg/mL) group. Data are normalized to the *RN18S* gene expression. Asterisks indicate statistical significance (^*^p < 0.05 and ^**^p < 0.01). For all graphs, the value represents mean ± SEM. The experiments are replicated three or four times.

After determining that the optimal Myo-Ins supplementation concentration during the liquid preservation of boar semen was 2 mg/mL, we conducted qRT-PCR using the control sample and 2 mg/mL on day 7 samples. The expression levels of *NRF2* and *GCLC* were significantly increased (*p* < 0.05) in the 2 mg/mL sample compared to those in the control sample. In contrast, the expression levels of *NQO1*, *KEAP1*, and *GSR* were not significantly different from those in the control sample (Figure 6).

## 4. Discussion

During liquid preservation of boar semen, spermatozoa are susceptible to oxidative stress, and antioxidants are added to the extender to decrease the detrimental effects of ROS levels on sperm quality (30, 31). Myo-inositol is a type of vitamin B1 acting as a strong antioxidant, and its concentration in the boar reproductive tract is 1–2 mM (19); however, previous studies demonstrated that the *in vitro* optimal concentration of Myo-Ins in humans is 2–20 mg/mL (32), in bulls (3 mg/mL) (24), and in dogs (1 mg/mL) (1) therefore, in this study, we used 2, 4, 6, and 8 mg/mL Myo-Ins. To date, the impact of Myo-Ins supplementation on boar semen quality during liquid preservation has not been investigated. Therefore, this study aimed to evaluate the effect of different concentrations of Myo-Ins supplementation in extender on boar semen quality during liquid preservation by examining sperm function quality parameters such as kinematic parameters (33, 34), acrosome integrity, plasma membrane integrity, viability (35, 36), MMP (37, 38), and gene expression.

Sperm motility is a crucial factor in determining the effects of semen storage, and declines with longer storage duration (39). Sperm motility is an indicator of normal metabolism and intact membranes (39). Oxidative stress is known to reduce sperm motility and impair sperm function (40). Previous studies have shown that antioxidants protect sperm cells and support the maintenance of sperm motility during the preservation phase (31, 41). In previous studies, the beneficial effects of Myo-Ins on the motility of spermatozoa were elucidated by its ROS scavenging effect, thus controlling oxidative stress in mammalian sperm cells (42). In this study, sperm motility was evaluated on days 0, 1, 3, 5, and 7 in all the samples. These results indicated that sperm motility in the Myo-Ins-treated samples was higher than that in the control sample. Sperm kinematics parameters such as VCL, VAP, and VSL were higher at 4 mg/mL compared to the control group. In contrast, total motility and progressive motility were higher in the 2 mg/mL treated group compared to the 4 mg/mL group. The results of the present study also suggest that the treatment samples had a higher effective survival time than the control samples. These findings were similar to those of previous studies in which Myo-Ins was added to the freezing extender for sperm cryopreservation in dogs (1) and bulls (24). Furthermore, the optimum concentration for Myo-Ins was 2 mg/mL in the semen extender, in contrast to previous studies (1, 24). This might be due to the different animals, types of extenders, and preservation procedures. Concurrently, different temperatures for semen preservation may have also led to varying optimum concentrations of Myo-Ins, resulting in a variation from previous studies. These findings, along with the present data, suggest that a Myo-Ins supplemented extender may improve boar semen motility by reducing the negative effects of ROS produced by sperm cells during liquid preservation (1, 24).

The structure and functional integrity of the plasmalemma, which is the outer membrane of sperm, is important for the metabolism of sperm, ova binding, capacitation, and acrosome reaction (43). The integrity of the plasma membrane of sperm is pivotal for its survival inside the female reproductive tract (43). Damage to the plasmalemma can cause cellular death and loss of homeostasis (44). In the present study, HOST was used to investigate sperm plasma membrane integrity in the control and Myo-Ins-treated samples during semen preservation. The number of HOS-positive spermatozoa was higher on days 5 and 7 at 2 mg/mL samples compared to the control samples, which is similar to the plasma membrane integrity result of dog cryopreserved sperm, in which a high intact plasma membrane was observed in the treatment samples (1). On the other hand, sperm viability, a vital sperm quality parameter, was assessed in the control and treatment samples, and showed more viable spermatozoa in the treated samples, which is in accordance with previous studies on human and dog sperm where high levels of viable spermatozoa were seen in Myo-Ins-treated samples (1, 45). The plasma membrane integrity and sperm viability were higher at 4 mg/mL on day 1 compared to the 2 mg/mL sample. Although the result of sperm viability on day 3 is numerically higher than that of day1, it is difficult to say that it has increased because it is within the error range.

Acrosome integrity is a key indicator of sperm quality (46). The acrosome is an essential organelle that makes it easier for spermatozoa to pass through the oocyte’s zona pellucida before fertilization (46). In this study, Myo-Ins supplementation improved sperm motility, membrane integrity, and acrosome integrity, but there was a difference between high and low dose levels that resulted in these parameters being lower than in the control group. A Myo-Ins addition of 2 mg/mL proved to be the optimal concentration in boar semen liquid preservation. Therefore, we assume that Myo-Ins is a dose-dependent chemical and high concentration may reduce the sperm quality parameters in boar. Our results also showed that 2 mg/mL Myo-Ins treatment of extender exerted a protective effect on acrosome integrity in boar semen liquid preservation. These findings are similar to those of Qamar et al. (1) where 1 mg/mL Myo-Ins resulted in a higher percentage of intact sperm acrosomes in dogs after freeze-thawing; similarly, in the present study, we found that the percentage of intact acrosomes was higher at 2 mg/mL Myo-Ins on day 7 compared to the control group. This result indicates that the long-term storage of boar semen with Myo-Ins may improve the quality of sperm cells and cause less damage to the acrosome.

Oxidative stress plays a major role in the deterioration of boar semen quality during liquid preservation (47). Previous studies have shown that the sperm plasma membrane of mammals is rich in unsaturated fatty acids and thus susceptible to peroxide damage (48). During preservation, sperm-generated ROS (lipid hydroperoxides, hydrogen peroxides, and peroxide anion free radicals) induce damage to spermatozoa, resulting in decreased sperm quality (49). In this study, we observed that the accumulation of sperm ROS during liquid preservation occurred and subsequently led to oxidative stress in sperm resulting in decreased sperm motility, acrosome integrity and viability which are considered as the most sensitive indicators of ROS (6, 50). On the basis of these sperm ROS indicators, we assume that oxidative stress decreased during the *in vitro* storage of sperm Therefore, in order to improve the semen quality antioxidants are added to improve the semen quality and decrease the oxidative stress. It has been recommended that Myo-Ins is a strong antioxidant and ROS scavenger, which consequently improves sperm quality. Therefore, adding Myo-Ins to the extender improved semen quality by attenuating ROS stress, which is consistent with previous studies on human and dog sperm (1, 42, 51). Correspondingly, we analyzed the relative expression of antioxidant related genes (*NRF2, NQO1, GCLC, GSR,* and *KEAP1*) in the control and 2 mg/mL Myo-Ins treatments. Since all the sperm quality parameters were best seen in the 2 mg/mL of Myo-Ins therefore, we selected 2 mg/mL as the optimal concentration to perform gene expression, which is similar to the previous studies on humans, improving and enhancing the sperm quality parameters with antioxidant capacity (12, 52). The activity of antioxidant genes, such as *NRF2* and *GCLC* significantly increased with the addition of an optimal concentration of Myo-Ins, which is similar to previous findings in fish (53). In contrast, the expression levels of *NQO1, GSR* and *KEAP1* were increased but not significantly compared to those in the treatment sample. Taken together, these findings suggest that the addition of Myo-Ins to the extender may improve boar semen quality by ameliorating ROS levels during storage.

Mitochondria are vital organelles and involved in redox regulation, ATP synthesis, and apoptosis (54). Interestingly, Condorelli et al. (18) investigated the role of Myo-Ins in sperm mitochondria, where they found an increased number of high membrane potential spermatozoa in OAT patients, which is in line with this study in which high membrane potential spermatozoa were found in Myo-Ins treated samples in a time-dependent manner during liquid preservation. Myo-Ins acts specifically at the level of mitochondria exerting positive effects on the ATP production via oxidative phosphorylation process subsequently results in high sperm motility, viability and MMP In this study, we assumed that Myo-Ins might protect boar semen against ROS by inhibiting apoptosis and inducing protection of sperm mitochondria; however, further studies are required to evaluate the specific mechanisms of Myo-Ins protection against mitochondria (18). We may hypothesize that Myo-Ins acts as an antioxidant and improves sperm motility and assesses plasma membrane integrity, which is related to the antioxidant content and high sperm viability.

The present study has some limitations in that the level of ROS was not determined using commercial kits such as MDA, ROS, and total antioxidant capacity kits (55). Secondly, we chose only one dose, i.e., 2 mg/mL since all the sperm parameters were best observed and the control group to investigate the antioxidant gene expression. Further study is required to investigate the effect of Myo-Ins on boar semen during liquid preservation to overcome these limitations.

## 5. Conclusion

The present study shows that the addition of Myo-Ins to the semen extender improved sperm motility, viability, plasma membrane integrity, acrosome integrity, and MMP and significantly improved the antioxidant related genes (*NRF2, GCLC*) in pigs. Additionally, 2 mg/mL was determined to be the optimal of Myo-Ins concentration for liquid preservation. These data could facilitate the development of a strategy for liquid storage of boar semen.

## Data availability statement

The original contributions presented in the study are included in the article, further inquiries can be directed to the corresponding authors.

## Author contributions

AJ, JL, and S-HH: conceptualization, validation, writing—(original draft preparation), and writing—(review and editing). AJ, DO, HC, MK, and LC: methodology, investigation, and formal analysis. JL and S-HH: funding acquisition. All authors contributed to the article and approved the submitted version.

## Funding

This work was supported, in part, by a grant from the “National Research Foundation of Korea grant funded by the Korean Government (2020R1A2C2008276 and 2021R1C1C2013954),” “Regional Innovation Strategy (RIS) through the National Research Foundation of Korea (NRF) funded by the Ministry of Education (MOE) (2021RIS-001),” and “Korea Institute of Planning and Evaluation for Technology in Food, Agriculture, Forestry and Fisheries (IPET) through Agriculture, Food and Rural Affairs Convergence Technologies Program for Educating Creative Global Leader (grant number: 320005-4), funded by Ministry of Agriculture, Food and Rural Affairs (MAFRA),” Republic of Korea.

## Conflict of interest

The authors declare that the research was conducted in the absence of any commercial or financial relationships that could be construed as a potential conflict of interest.

## Publisher’s note

All claims expressed in this article are solely those of the authors and do not necessarily represent those of their affiliated organizations, or those of the publisher, the editors and the reviewers. Any product that may be evaluated in this article, or claim that may be made by its manufacturer, is not guaranteed or endorsed by the publisher.
